# Study on anxiety and depression of men who have sex with men: An application of group-based trajectory model

**DOI:** 10.3389/fpsyg.2022.857203

**Published:** 2023-01-03

**Authors:** Dan Wu, Xiaoni Zhong, Ruibin Deng, Hong Pan, Yuwen Gao, Bing Lin, Xian Tang, Jianghong Dai, Hao Liang, Ailong Huang

**Affiliations:** ^1^Research Center for Medicine and Social Development, Chongqing Medical University, Chongqing, China; ^2^Innovation Center for Social Risk Governance in Health, Chongqing Medical University, Chongqing, China; ^3^Changzhou Center for Disease Control and Prevention, Changzhou, China; ^4^Department of Epidemiology and Health Statistics, School of Public Health, Xinjiang Medical University, Xinjiang, China; ^5^Department of Epidemiology and Health Statistics, School of Public Health, Guangxi Medical University, Nanning, China; ^6^Key Laboratory of Molecular Biology, Ministry of Molecular Biology, Chongqing, China

**Keywords:** PrEP, anxiety, depression, group-based trajectory model, MSM

## Abstract

The prevalence of anxiety and depression is high among men who have sex with men (MSM), but limited studies focus on their development trends. This study examined the prevalence and influencing factors of anxiety and depression in HIV-negative MSM. In this study, 711 subjects were followed up every 24 weeks for 96 weeks. A group-based trajectory model was used to identify different development trends and a logistic regression model was used to explore the influencing factors of different trajectories. Low, moderate, and high anxiety groups accounted for 32.56, 56.12, and 11.32%; and low and high depression groups accounted for 73.90 and 26.10%. High anxiety was associated with a lower education degree, little HIV-related knowledge, and other characteristics (more agreed that “HIV is a threat to me/my family,” had high frequency of seeking sexual partners *via* the Internet, had a history of sexually transmitted disease (STD), and a sense of discrimination by doctors). High depression was associated with a lower education degree, little HIV-related knowledge, being bisexual and the following characteristics: they felt that HIV infection rate of MSM around them was high, and more agreed that “HIV is a threat to me/my family,” had a high frequency of seeking sexual partners through the Internet and alcohol drinking, had a history of STD, and felt being discriminated against by doctors and others. There is heterogeneity in the development trends of anxiety and depression. We need to improve intervention in the mental health of MSM, especially those with high anxiety and depression development trends.

**Clinical Trial Registration:**

[http://www.chictr.org.cn/showproj.aspx?proj=5716], identifier [ChiCTR-TRC-13003849].

## Introduction

Although great progress has been made in the prevention of human immunodeficiency virus (HIV), the continued prevalence of HIV still exists (Korenromp et al., 2015). According to the latest statistics of the joint United Nations Program on HIV/AIDS (UNAIDS, 2019), the number of new HIV infections in the world decreased from 2.10 million in 2010 to 1.70 million in 2018, a decrease of 16%, but it is still far from the target of less than 500,000 by 2020. In China, the overall epidemic situation of HIV shows a low level of prevalence. By the end of October 2019, 958,000 people were reported to be living with HIV, among whom 131,000 people were newly infected. MSM accounted for 23.0% in those new infections (He, 2019). Owing to physiological, socio-psychological, structural, and other factors, the risk of HIV infection in MSM is more than ten times higher than that of the general population (Chen and Ma, 2018), and it is also the most rapid population of HIV infection in China at present. Several studies have pointed out that high-risk behavior and HIV infection are related to psychological problems, such as anxiety and depression (Hanrahan et al., 2011; Pappin et al., 2012; Hill et al., 2018; Rogers et al., 2018; Miller et al., 2021). At present, most HIV prevention strategies for MSM are behavioral interventions, such as strengthening the condom use, promoting pre-exposure prophylaxis (PrEP), encouraging regular HIV testing, and consultation, which ignore the effect of psychological intervention in HIV prevention and control. Meanwhile, due to the special sexual orientation and concept of traditional Chinese culture, MSM in China experience disproportionately burdensome degrees of stigma, prejudice, and discrimination (Steward et al., 2013). Social exclusion and discrimination, negative stereotypes of internalization, and lack of support often make MSM conceal their sexual orientation and bear long-term psychological burden, thus leading to a higher prevalence of anxiety and depression than the general population (Hu et al., 2015). Studies have shown that mental disorders, such as depression and anxiety, contribute to HIV vulnerability and suicidal ideation (Yu et al., 2018; Zhang et al., 2019; Storm et al., 2021). Further, it will lead to the increase of new HIV infections, hence we ought to focus on the occurrence and development of anxiety and depression in this population.

To date, there are limited longitudinal studies of anxiety and depression in the HIV-negative MSM population. Previous studies have typically focused on anxiety and depression at a certain time point, while ignoring the dynamic changing characteristics in psychology. This study aimed to identify the development trend of anxiety and depression with a group-based trajectory model and discuss the influencing factors for different development trends. Therefore, we could accordingly conduct targeted intervention for individuals with high anxiety and depression development trajectories in the future, which can provide the theoretical basis and guidance for the subsequent prevention and intervention policies formulation of HIV-negative MSM. It also provides a basis for formulating prevention and intervention policies and guidelines for the mental health of HIV-negative MSM. Furthermore, it may also indirectly help to reduce new HIV infections.

## Methods

### Study design and participants

The study was a subset analysis of an open, non-randomized, multicenter, parallel-controlled clinical intervention trial based on standard HIV prevention interventions (registration number: ChiCTR-TRC-13003849; Ethical Approval code: 2012010, approved at 2012.4.5). HIV-negative MSM were recruited by non-probability sampling. The inclusion criteria of this study were: (1) being between the ages of 18 and 65 years; (2) had at least once sex every 2 weeks; (3) had at least one same-sex partner; (4) being willing to participate in the trial for 96 weeks; and (5) signed the informed consent. Exclusion criteria: no anxiety or depression follow-up information (3 or more missed visits). Inclusion criteria 2 and 3 were decided based on the overall research project, and this exclusion criterion was added posteriorly to ensure the power of the statistical analysis.

All eligible subjects in this study were under 96-week cohort management and were followed up face-to-face every 24 weeks to collect information on anxiety and depression through self-completed questionnaires.

### Measures

The following information was mainly collected in the baseline survey:

(1) Demographic characteristics: age, nationality, degree of education, employment status, marital status, monthly income, etc.

(2) Psychosocial characteristics: sexual orientation, sexual partners’ attitude to PrEP, level of fear of discrimination by others, felt being discriminated against by doctors, felt trusted by doctors, etc.

(3) HIV-related knowledge, attitude, and behavior: 13 HIV-related questions (for example, correct use of condom during every insertional sex can avoid HIV infection, and the options are correct, incorrect and do not know), with 1 point for a correct answer and 0 points for the rest. It also included questions on HIV-related attitudes and behaviors, such as views on the severity of HIV, history of HIV testing, history of HIV free consultation, and frequency of seeking sexual partners through the Internet.

(4) Anxiety and depression measurement. Anxiety was measured using the Self-Rating Anxiety Scale (Zung, 1971). Several studies have proven its substantial reliability and validity. It can accurately reflect the subjective anxiety feelings of the participants. The scale used a 4-point scale with a total of 20 items, including 15 positive items (1–4) and 5 negative items (4–1). The scores of 20 items were aggregated as raw scores, then multiplied by 1.25 as the standard score. A standard score greater than or equal to 50 indicates anxiety. The higher the score, the more serious the symptom was (50–59 points for mild, 60–69 points for moderate, 70 points or more for severe anxiety). Depression was measured using the Center for Epidemiologic Studies Depression Scale (CES-D), which is currently widely used in the international depression screening (Park and Yu, 2021). It consisted of 16 forward and four reverse items. Each item ranged from 1 (occasionally or no) to 4 (most of the time). When the scoring standard was 0–3 for each item, an aggregate score greater than or equal to 16 indicated depression. The higher the score, the more serious the symptom was. Both scales were proved to have fine reliability and validity, their Cronbach’s α values for both were >0.8 according to the relevant literature (Samakouri et al., 2012; Yu et al., 2015; Ilic et al., 2019; Jiang et al., 2019). The Chinese version of SAS and CES-D used in the present study had acceptable internal consistency (the average values of Cronbach’s α coefficient were 0.785 and 0.766 separately).

### Statistical analysis

Epidata3.1 software was used for data double entry and verification, and all statistical analyses were conducted using SAS9.4 software. (1) The distribution of categorical variables was represented by frequency and rate, and the difference analysis was performed by the χ^2^ test; the distribution of continuous variables was described using the mean ± standard deviation (SD), and the difference analysis was performed by the *t*-test or ANOVA. (2) Group-based trajectory models were fitted in the order of 1–4 subgroups, with the highest-order parameters in each subgroup guaranteed to be statistically significant and the least linear parameters retained if not (Berlin et al., 2014; Feng et al., 2014). The Bayesian information criterion (BIC) and average posterior probability (AvePP) were the indicators of fit evaluation (Nagin and Tremblay, 2001; Jones and Nagin, 2007; Lin et al., 2017), and BIC close to 0 and AvePP > 0.7 indicated that the model fit well, and the optimal number of trajectory groups was selected according to the above indicators. (3) In the trajectory grouping effect analysis, the group of anxiety and depression was an independent variable separately in the generalized estimating equation, and the grouping effect was indirectly evaluated by whether the grouping factor was statistically significant. (4) In the analysis of the influence factors of the anxiety and depression trajectory groups. Univariate analysis was performed using the χ^2^ test and the *t*-test or ANOVA, and variables with *p* < 0.150 were included in the multivariate stepwise logistic regression (the entry and exclusion criteria were 0.10 and 0.15). All variables were statistically significant at *p* < 0.050. In addition, although the trajectory model and the generalized estimating equation have certain robustness to the data with missing values (Nelder and Wedderburn, 1972; Nagin and Odgers, 2010), the parameter estimates were significantly biased when the missing values were high. Therefore, MSM who were missing three or more times at follow-up were excluded from the analysis.

## Results

### Subject characteristics

**FIGURE 1:**
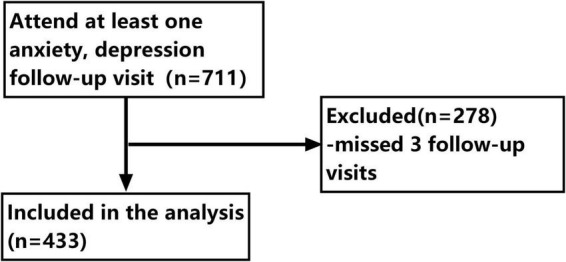
Flow diagram for the clinical trial.

In total, 2,422 MSM were recruited across the four research centers. According to the inclusion and exclusion criteria, 711 MSM were included in the study, and 278 MSM were excluded from the study for three missed follow-ups, and 433 MSM were included in the analysis. The differences on age, ethnicity, education level, employment status, marital status, and monthly income between 278 excluded and 433 included were not statistically significant (*p* > 0.050; as shown in details in Table 1 and Figure 1).

**TABLE 1:** Comparisons of demographic characteristics between the inclusion group and the exclusion group among men who have sex with men (MSM).

Variable	Characteristic	Inclusion group (%)	Exclusion group (%)	χ^2^ value	*P*
Age	18–25	150 (36.64)	96 (34.53)	1.04	0.595
	26–35	167 (38.57)	116 (41.73)		
	>36	116 (26.79)	66 (23.74)		
Nationality*	Han nationality	385 (88.91)	256 (92.09)	1.92	0.166
	Ethnic minority	48 (11.09)	22 (7.91)		
Degree of Education*	Junior high school or below	47 (10.90)	30 (10.79)	1.71	0.425
	Senior high school/vocational high school/technical secondary school	105 (24.36)	77 (27.70)		
	Junior college, college or above	279 (64.73)	171 (61.51)		
Employment status*	Employed	348 (80.56)	218 (78.42)	0.64	0.725
	Students	49 (11.34)	37 (13.31)		
	Unemployed or retired	35 (8.10)	23 (8.27)		
Marital status	Unmarried	320 (73.90)	208 (74.82)	0.76	0.685
	Married	69 (15.94)	47 (16.91)		
	Divorced or widowed	44 (10.16)	23 (8.27)		
Monthly income (yuan)*	≤1,000	57 (13.35)	46 (16.73)	3.17	0.366
	1,001–3,000	150 (35.13)	103 (37.45)		
	3,001–5,000	165 (38.64)	90 (32.73)		
	≥5,001	55 (12.88)	36 (13.09)		

*Missing data.

### Group-based trajectory model

The number of trajectories of anxiety and depression were fitted to trajectories of 1–4 in order. Since the dependent variable is the total score of the anxiety and depression scales, both of which were approximately normal distributions, a censored normal model was used in the trajectory model. The subgroup 1 did not have trajectory discrimination and it was therefore for information only. Table 2 shows the BIC and AvePP (min-max) for each subgroup. For anxiety, the BIC was −5,431.61 with AvePP both >0.7. When the subgroup number was 3, its BIC was closer to 0 compared with 1 and 2 subgroups. When the subgroup number was 4, there was no statistically significant linear parameter for the fourth subgroup, although the BIC was closer to 0 compared with 3 subgroups. For depression, when the subgroup number was 2, its BIC was slightly lower than 3 and 4 subgroups, however, there was no statistically significant linear parameter for the first subgroup when the subgroup number was 3. Considering the principle of model simplification, the same higher-order parameters should be statistically significant, the optimal number of subgroups was 3 and 2 for anxiety and depression, respectively.

**TABLE 2:** Comparison of the model fit index between different subgroup number in anxiety and depression among men who have sex with men (MSM).

Variable	Subgroup number	BIC	AvePP			
			1	2	3	4
Anxiety	1	−5,631.40	1.00 (1.00–1.00)	–	–	–
	2	−5,501.46	0.91 (0.53–1.00)	0.86 (0.50–1.00)	–	–
	3	−5,431.61	0.90 (0.51–1.00)	0.90 (0.52–1.0)	0.89 (0.51–1.00)	–
	4	−5,423.83	0.92 (0.51–1.00)	0.88 (0.44–1.00)	0.79 (0.49–1.00)	0.91 (0.47–1.00)
Depression	1	−5,533.21	1.00 (1.00–1.00)	–	–	–
	2	−5,421.00	0.93 (0.51–1.00)	0.87 (0.50–1.00)	–	–
	3	−5,367.01	0.89 (0.50–1.00)	0.89 (0.52–1.00)	0.88 (0.55–1.00)	–
	4	−5,342.71	0.93 (0.59–1.00)	0.86 (0.49–1.00)	0.76 (0.36–1.00)	0.88 (0.42–1.00)

**FIGURE 2:**
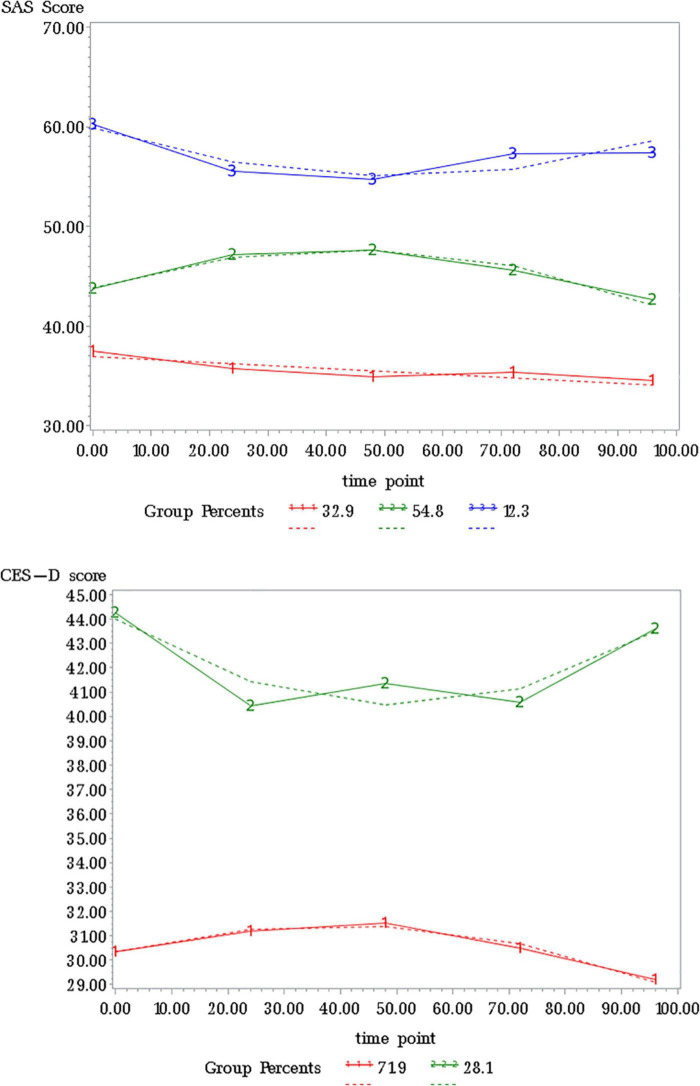
An optimal trajectory model curve of anxiety and depression.

Table 3 and Figure 2 showed the detailed results of the best grouping results. On anxiety, the first, second, and third subgroup accounted for 32.56, 56.12, and 11.32%, respectively. On depression, subgroup 1 and 2 accounted for 73.90 and 26.10%, separately.

**TABLE 3:** The parameters of the optimal trajectory model among men who have sex with men (MSM).

Variable	Subgroup number	Parameter	Estimates	Standard error	*T*	*P*
Anxiety	1	Intercept	36.97	0.61	60.68	0.000
		Linear	−0.03	0.01	−2.64	0.008
	2	Intercept	43.84	0.64	68.89	0.000
		Linear	0.18	0.03	5.75	0.000
		Quadratic	−0.01	0.01	−5.75	0.000
	3	Intercept	59.89	1.30	45.94	0.000
		Linear	−0.19	0.06	−3.17	0.002
		Quadratic	0.01	0.01	2.90	0.004
Depression	1	Intercept	30.33	0.59	51.79	0.000
		Linear	0.06	0.02	2.35	0.019
		Quadratic	−0.01	0.01	−2.69	0.007
	2	Intercept	44.02	1.45	30.43	0.000
		Linear	−0.14	0.06	−2.50	0.012
		Quadratic	0.01	0.01	2.19	0.028

### Grouping effectiveness evaluation

The specific levels of anxiety and depression scores at baseline and 4 follow-up time points in different trajectory subgroups are shown in Table 4. The generalized estimating equations were used to evaluate the trajectory grouping effects of anxiety and depression. The results showed that the χ^2^ values of the anxiety and depression grouping effects were 201.61 and 119.27, respectively, with *p* < 0.05. The Bonferroni method was used for multiple comparisons of different trajectory groups at the same follow-up time point. Multiple comparisons of total anxiety and depression scores at baseline and at the four follow-up time points were statistically significant. This can be inferred indirectly that the trajectory analysis model grouping is better and can effectively distinguish the anxiety and depression trends across trajectory groups. In addition, the prevalence of anxiety and depression was higher at baseline and at each follow-up time point, with the prevalence of anxiety ranging from 24.70 to 27.92% and the prevalence of depression ranging from 33.33 to 48.22%, with a higher prevalence of depression than anxiety in the MSM population.

**TABLE 4:** Comparison of the subgroups of anxiety and depression at each follow-up point.

Variable	Subgroup	Baseline	24th week	48th week	72th week	96th week
Anxiety	Low	37.57 ± 7.63*^a^*	35.17 ± 5.06*^a^*	34.36 ± 4.90*^a^*	34.88 ± 4.97*^a^*	34.13 ± 5.34*^a^*
	Moderate	43.76 ± 8.29*^b^*	47.38 ± 5.16*^b^*	47.98 ± 5.82*^b^*	45.70 ± 7.01*^b^*	43.47 ± 7.09*^b^*
	High	60.89 ± 8.71*^c^*	56.29 ± 8.53*^c^*	55.29 ± 6.47*^c^*	58.88 ± 7.00*^c^*	58.16 ± 5.80*^c^*
Incidence rate		26.39%	27.71%	27.92%	24.70%	25.00%
Depression	Low	30.23 ± 6.99*^a^*	31.23 ± 6.63*^a^*	31.55 ± 7.60*^a^*	30.63 ± 6.60*^a^*	29.47 ± 5.96*^a^*
	High	45.63 ± 9.03*^b^*	40.96 ± 6.80*^b^*	41.89 ± 7.03*^b^*	41.48 ± 8.30*^b^*	44.67 ± 11.07*^b^*
Incidence rate		39.03%	45.78%	48.22%	37.25%	33.33%

^*a*–*c*^No statistical difference between groups with the same letter at the time point.

### Influencing factors of anxiety and depression

#### Univariate analysis

Demographic characteristics, psychosocial characteristics, HIV-related knowledge, attitude, and behavior were included in the univariate analysis (Table 5). In terms of anxiety, on demographic characteristics, education degree was a significant variable, subjects with junior high school or below education degree were significantly more in the high anxiety group. On HIV-related knowledge, attitude and behavior, scores of HIV-related knowledge were significant different in low, moderate, and high anxiety subgroups; history of HIV test or HIV free consultation did not differ significantly between the subgroups; MSM with a history of STD in the low anxiety subgroup were significantly fewer. On psychosocial characteristics, MSM who felt being discriminated against by doctors showed significant differences between the three subgroups of anxiety.

**TABLE 5:** A univariate analysis of anxiety and depression.

Variable	Low anxiety (*n* = 141,%)	Moderate anxiety (*n* = 243, %)	High anxiety (*n* = 49, %)	χ^2^/*F*	*P*	Low depression (*n* = 320, %)	High depression (*n* = 113, %)	χ^2^/*T*	*P*
Age (years)				5.20	0.268			8.60	0.014
18–25	40 (28.37)	88 (36.21)	22 (44.90)			101 (31.56)	49 (43.36)		
26–35	61 (43.26)	91 (37.45)	15 (30.61)			136 (42.50)	31 (27.43)		
>36	40 (28.37)	64 (26.34)	12 (24.49)			83 (25.94)	33 (29.21)		
Nationality				1.61	0.447			0.03	0.854
Han nationality	128 (90.78)	212 (87.24)	45 (91.84)			284 (88.75)	101 (89.38)		
Ethnic minority	13 (9.22)	31 (12.76)	4 (8.16)			36 (11.25)	12 (10.62)		
Degree of Education*				11.60	0.021			14.10	0.001
Junior high school or below	8 (5.71)	28 (11.57)	11 (22.45)			25 (7.86)	22 (19.47)		
Senior high school/vocational high school/technical secondary school	35 (25.00)	57 (23.55)	13 (26.53)			74 (23.27)	31 (27.43)		
Junior college, college or above	97 (69.29)	157 (64.88)	25 (51.02)			219 (68.87)	60 (53.10)		
Employment status*				4.58	0.333			6.53	0.038
Employed	116 (82.27)	197 (81.40)	35 (71.43)			266 (83.39)	82 (72.57)		
Students	17 (12.06)	25 (10.33)	7 (14.29)			32 (10.03)	17 (15.04)		
Unemployed or retired	8 (5.67)	20 (8.26)	7 (14.29)			21 (6.58)	14 (12.39)		
Marital status				1.82	0.768			0.80	0.669
Unmarried	106 (75.18)	178 (73.25)	36 (73.47)			239 (74.69)	81 (71.68)		
Married	20 (14.18)	39 (16.05)	10 (20.41)			48 (15.00)	21 (18.58)		
Divorced or widowed	15 (10.64)	26 (10.70)	3 (6.12)			33 (10.31)	11 (9.73)		
Monthly income (yuan)*				5.77	0.449			3.37	0.338
<1,000	18 (12.86)	33 (13.81)	6 (12.50)			38 (12.06)	19 (16.96)		
1,001–3,000	53 (37.86)	76 (31.80)	21 (43.75)			107 (33.97)	43 (38.39)		
3,001–5,000	47 (33.57)	101 (42.26)	17 (35.42)			127 (40.32)	38 (33.93)		
>5,001	22 (15.71)	29 (12.13)	4 (8.33)			43 (13.65)	12 (10.71)		
Score of HIV-related knowledge				12.93	0.000			4.03	0.000
	10.16 ± 2.08	9.64 ± 2.43	8.10 ± 3.30			9.95 ± 2.28	8.74 ± 2.87		
HIV test				0.92	0.630			0.24	0.624
Yes	115 (81.56)	205 (84.36)	39 (79.59)			267 (83.44)	92 (81.42)		
No	26 (18.44)	38 (15.64)	10 (20.41)			53 (16.56)	21 (18.58)		
HIV free consultation *				4.35	0.114			5.35	0.021
Yes	105 (75.54)	166 (68.60)	29 (60.42)			232 (72.96)	68 (61.26)		
No	34 (24.46)	76 (31.40)	19 (39.58)			86 (27.04)	43 (38.74)		
Views on the severity of HIV*				11.74	0.019			0.79	0.675
Very serious	86 (60.99)	178 (73.25)	31 (64.58)			215 (67.19)	80 (71.43)		
Serious	45 (31.94)	57 (23.46)	11 (22.92)			86 (26.88)	27 (24.11)		
General and below	10 (7.09)	8 (3.29)	6 (12.50)			19 (5.94)	5 (4.46)		
HIV infection rate of MSM around*				1.12	0.892			7.87	0.020
Very high	34 (24.11)	63 (26.03)	14 (29.17)			71 (22.26)	40 (35.71)		
High	66 (46.81)	113 (46.69)	19 (39.58)			153 (47.96)	45 (40.18)		
General and below	41 (29.08)	66 (27.27)	15 (31.25)			95 (29.78)	27 (24.11)		
The threat of HIV to yourself/family*				12.14	0.016			5.17	0.076
Very serious	71 (50.35)	149 (61.57)	31 (64.58)			179 (56.11)	72 (64.29)		
Serious	28 (19.89)	55 (22.73)	7 (14.58)			65 (20.38)	25 (22.32)		
General and below	42 (29.79)	38 (15.70)	10 (20.83)			75 (23.51)	15 (13.39)		
sexual orientation*				0.41	0.816			2.36	0.125
With men only	49 (34.75)	83 (34.16)	14 (29.79)			115 (35.94)	31 (27.93)		
With men and women	92 (65.25)	160 (65.84)	33 (70.21)			205 (64.06)	80 (72.07)		
Frequency of seeking sexual partners through Internet*				7.90	0.095			9.55	0.008
Often	5 (3.70)	21 (8.97)	7 (15.22)			17 (5.56)	16 (14.68)		
Sometimes or occasional	86 (63.70)	132 (56.41)	23 (50.00)			185 (60.46)	56 (51.38)		
None	44 (32.59)	81 (34.62)	16 (34.78)			104 (33.99)	37 (33.94)		
Frequency of drinking alcohol*				6.67	0.155			9.57	0.008
At least 3 times/week	15 (10.64)	31 (12.81)	12 (25.00)			34 (10.66)	24 (21.43)		
About once/week	81 (57.45)	133 (54.96)	22 (45.83)			185 (57.99)	51 (45.54)		
None	45 (31.91)	78 (32.23)	14 (29.17)			100 (31.35)	37 (33.04)		
History of STD*				7.69	0.021			3.24	0.072
Yes	6 (4.26)	31 (12.81)	6 (12.77)			27 (8.46)	16 (14.41)		
No	135 (95.74)	211 (87.19)	41 (87.23)			292 (91.54)	95 (85.59)		
Sexual partners’ attitude on PrEP*				7.32	0.120			4.68	0.097
Support	51 (36.17)	113 (47.28)	19 (40.43)			144 (45.43)	39 (35.45)		
Object	13 (9.22)	13 (5.44)	6 (12.77)			20 (6.31)	12 (10.91)		
Indifferent	77 (54.61)	113 (47.28)	22 (46.81)			153 (48.26)	59 (53.64)		
worried that the medicine does not work*				1.12	0.891			5.14	0.076
A little or none	70 (50.00)	113 (46.69)	23 (46.94)			159 (50.00)	47 (41.59)		
Somewhat	43 (30.71)	72 (29.75)	16 (32.65)			98 (30.82)	33 (29.20)		
Majority and above	27 (19.29)	57 (23.55)	10 (20.41)			61 (19.18)	33 (29.20)		
Worried about being discriminated against by others because of PrEP*				1.65	0.799			5.70	0.058
A little or none	71 (50.71)	126 (52.07)	24 (48.98)			173 (54.40)	48 (42.48)		
Somewhat	27 (19.29)	56 (23.14)	11 (22.45)			68 (21.38)	26 (23.01)		
Majority and above	42 (30.00)	60 (24.79)	14 (28.5)			77 (24.21)	39 (34.51)		
Felt being discriminated against by doctors*				6.58	0.037			8.11	0.004
No	129 (91.49)	208 (85.60)	38 (77.55)			286 (89.38)	89 (78.76)		
Yes	12 (8.51)	35 (14.40)	11 (22.45)			34 (10.63)	24 (21.24)		
Felt trusted on doctors*				8.45	0.076			5.17	0.076
A little or none	15 (10.71)	38 (15.70)	13 (26.53)			43 (13.52)	23 (20.35)		
Somewhat	11 (7.86)	18 (7.44)	1 (2.04)			26 (8.18)	4 (3.54)		
Majority and above	114 (81.43)	186 (76.86)	35 (71.43)			249 (78.30)	86 (76.11)		

*Missing data.

In terms of depression, on demographic characteristics: age, education degree, and employment status were significantly different between the low and high depression subgroups. On HIV-related knowledge, attitude, and behavior: subjects in the high depression subgroup had a lower HIV-related awareness then those in the low depression group; 72.96 and 61.26% of MSM had a history of HIV free consultation in the low and high depression groups, separately, which was a statistically significant variable; subjects in the high depression subgroup had a higher frequency of seeking sexual partners through the Internet and alcohol drinking than those in the low depression subgroup. On psychosocial characteristics: MSM who felt being discriminated against by doctors were significantly more in the high depression group as shown in details in Table 5).

#### Multivariate logistic regression analysis

An ordinal logistic stepwise regression model was used to explore the influencing factors of anxiety, in which low anxiety was taken as a reference group. The results showed that the likelihood ratio χ^2^ of the model was 50.49, *p* = 0.000 < 0.050, and the model satisfied the principle of parallel line assumption (χ^2^ = 8.93, *p* = 0.444 > 0.050); also, Pearson’s goodness-of-fit χ^2^ was 622.59, *p* = 0.848 > 0.050, indicating a fine data fitting. The high anxiety was associated with education degree, HIV-related knowledge, HIV threat to themselves/family, frequency of seeking sexual partners through the Internet, history of STD, and doctor discrimination.

A binary logistic stepwise regression model was conducted to explore the influencing factors of depression, and low depression was taken as the reference. The results showed that the likelihood ratio χ^2^ of the model was 80.74, *p* = 0.000 < 0.050, and the Hosmer and Lemeshow goodness-of-fit χ^2^ was 5.10, *p* = 0.747 > 0.050, indicating a fine data fitting. High depression was associated with education degree, HIV-related knowledge, HIV infection rate of MSM around, HIV threat to themselves/family, sexual orientation, frequency of seeking sexual partners through the Internet, frequency of drinking alcohol, history of STD, worried about being discriminated against by others because of PrEP, and doctor discrimination. See Table 6 for details.

**TABLE 6:** Multivariate analysis of anxiety and depression among men who have sex with men (MSM).

Variable (reference group)	β	Waldχ^2^	*P*	*OR* (95%CI)
**Anxiety (low)**				
**Degree of Education (Junior high school or below)**				
Senior high school/vocational high school/technical secondary school	−0.80	4.31	0.038	0.45 (0.21–0.96)
Junior college, college or above	−0.84	5.51	0.019	0.43 (0.21–0.87)
Score of HIV-related knowledge	−0.14	10.44	0.001	0.87 (0.80–0.95)
**The threat of HIV to yourself/family (General and below)**				
Very serious	0.77	8.80	0.003	2.16 (1.30–3.58)
Serious	0.59	3.57	0.059	1.81 (0.98–3.35)
**Frequency of seeking sexual partners through Internet (Often)**				
Sometimes or occasional	−0.90	5.40	0.020	0.41 (0.19–0.87)
None	−0.69	2.92	0.088	0.50 (0.23–1.11)
History of STD (Yes)	−0.77	4.96	0.026	0.46 (0.23–0.91)
Felt being discriminated against by doctors (No)	0.80	6.94	0.008	2.22 (1.23–4.03)
**Depression (low)**				
**Degree of Education (Junior high school or below)**				
Senior high school/vocational high school/technical secondary school	−0.91	4.19	0.041	0.40 (0.17–0.96)
Junior college, college or above	−1.26	8.79	0.003	0.29 (0.12–0.65)
**Employment (Unemployed or retired)**				
Employed	−0.63	2.11	0.146	0.53 (0.23–1.25)
Students	0.37	0.47	0.491	1.45 (0.50–4.17)
Score of HIV-related knowledge	−0.19	11.89	0.001	0.83 (0.75–0.92)
**HIV infection rate of MSM around (General and below)**				
Very high	0.84	5.64	0.018	2.32 (1.16–4.66)
High	0.47	1.87	0.171	1.60 (0.82–3.13)
**The threat of HIV to yourself/family (General and below)**				
Very serious	0.99	5.93	0.015	2.69 (1.21–5.97)
Serious	1.26	7.24	0.007	3.51 (1.41–8.77)
Orientation (with men only)	0.65	5.13	0.034	1.93 (1.09–3.42)
**Frequency of seeking sexual partners through Internet (Often)**				
Sometimes or occasional	−1.17	7.13	0.008	0.309 (0.131–0.732)
None	−0.91	3.85	0.049	0.405 (0.164–0.999)
**Frequency of drinking alcohol (At least 3 times/week)**				
About once/week	−1.12	9.22	0.002	0.33 (0.16–0.67)
None	−0.89	4.96	0.026	0.41 (0.19–0.90)
History of STD (Yes)	−0.81	3.97	0.046	0.45 (0.20–0.99)
**Worried about being discriminated against by others because of PrEP (Majority and above)**				
A little or none	−0.73	5.40	0.020	0.48 (0.26–0.89)
Somewhat	−0.39	1.164	0.281	0.67 (0.33–1.38)
Felt being discriminated against by doctors (No)	0.84	5.51	0.019	2.33 (1.15–4.71)

## Discussion

Most of the current studies on anxiety and depression in the MSM population were cross-sectional, while anxiety and depression as psychological states were constantly changing and developing over time, traditional longitudinal studies tended to ignore the heterogeneity of individual development. In this study, the trajectory analysis model was used to group the anxiety and depression trends of MSM population, and according to the generalized estimating equations, the application of the trajectory analysis model had good generalizability. In addition, the prevalence of anxiety and depression was high among MSM in Western China. The prevalence of anxiety was maintained at 24.70–27.92% and depression was at 33.33–48.22% during the study, which was much higher than that of anxiety (1.32%) and depression (2.06%) in general populations (Zhang et al., 2019). Previous studies have shown that anxiety and depression in MSM significantly decrease HIV prevention effectiveness and treatment adherence (Gilman et al., 2001; Alvy et al., 2011). Prolonged anxiety and depression can seriously damage physical and mental health and can easily lead to disability, loss of life (Huang, 2013), and the substantial loss of total earnings and income (Hakulinen et al., 2019). Particularly, the COVID-19 pandemic has been reported to increase the prevalence of depressive symptoms substantially (Cerecero-Garcia et al., 2021). Psychological interventions for MSM need to be integrated into existing HIV prevention programs. Additionally, mechanisms to better monitor and diagnose mental health disorders are necessary.

The results of multivariate logistic regression analysis indicated that anxiety and depression in MSM were related to many factors. MSM with low education degree were more likely to have anxiety and depression, which is consistent with other current studies (Chen et al., 2003, 2019; Mu et al., 2016). The education degree is usually associated with cognitive ability and social status, MSM with low education degree are more vulnerable to Chinese mainstream culture due to poor understanding of HIV and subjective judgment. In addition, being discriminated by doctors was a risk factor for anxiety and depression, discrimination itself is likely to lead to the high incidence of MSM anxiety and depression (Xie et al., 2018). When MSM feels discriminated by doctors, it will aggravate the occurrence of anxiety and depression, seriously affect the doctor-patient relationship, and reduce MSM’s utilization of medical and health services and the efficiency of HIV prevention and control (Wight et al., 2006; Liu and Xu, 2008; Wang et al., 2010). In the follow-up interventions, first, we should change their disapproval on themselves, which includes strengthening the self-identity, building social network, and establishing positive values (Xu and Sheng, 2016); second, anti-discrimination education needs to be carried out for medical staff, good doctor-patient relationship is conducive to the relief of MSM’s anxiety and depression, such as strengthening the vocational and technical training of medical staff to be familiar with HIV prevention and treatment, and improving medical ethics (Qu et al., 2006). Third, strengthening the publicity and popularization of HIV among the public, creating a good social support environment (Han et al., 2018), considering family factors, helping MSM develop strategies for negotiating filial obligations (Steward et al., 2013), increasing the capabilities to buffer or cope with the unique marriage-related stressors (Yang et al., 2020), encouraging the establishment of MSM communities, and advocating MSM peer-lead interventions are helpful to address the high burden of mental health issues among MSM (Storm et al., 2021).

Anxiety and depression were also associated with HIV-related knowledge, attitude, and behavior. On HIV-related knowledge, a higher score was the protective factor. Lacking HIV-related knowledge will deepen MSM’s panic about HIV, which correspondingly leads to high anxiety and depression. HIV publicity and education has always been the traditional intervention measures for HIV prevention and control. The results of this study still proved the necessity of HIV health education. On HIV-related attitude, anxiety, and depression were higher in those who agreed that HIV was a threat to them/their family, and depression was higher in those who agreed that “The HIV infection rate of MSM around is very high.” It suggests that the risk perception of MSM is related to high anxiety and depression. Therefore, we should guide MSM to form a correct understanding of HIV, including transmission mode, infection risk, and treatment, to avoid creating excessive panic about HIV. For another, we should also encourage HIV-positive MSM to receive antiviral treatment and implement peer education, which helps other HIV-negative MSM relieve depression. In HIV-related behaviors, same as in the current studies, having a history of STD and seeking sexual partners online were risk factors for anxiety and depression (Zheng et al., 2005; Gao et al., 2012). An international survey showed considerable HIV prevalence and low awareness of PrEP among MSM who are able to use technology, have access to the Internet, and are probably users of social media websites and certain online applications (Pantavou et al., 2021). With the development of social media and machine learning techniques, negative emotions of MSM can be well monitored in an online and easy-to-use manner (Li et al., 2020). Therefore, targeted PrEP programs and psychological interventions could be conducted for potentially depressive MSM by an automated depressive emotion screening method. In addition, men who have sex with men and women (MSMW) were the high-risk group of depression compared with men who have sex with men only (MSMO), MSMW are more likely to be alienated and marginalized because of their sexual identity and sexual behavior, and suffer from discrimination of both heterosexual and homosexual populations (Stinchcombe et al., 2018; Bostwick et al., 2019; Friedman et al., 2019). In addition, their high gender role conflict may be a cause of mental health problems. We ought to strengthen the construction of social and mental health (Hu et al., 2019; Liu et al., 2020), help them improve their ability to regulate negative emotional states and maximize self-related positive emotions to alleviate depression among MSMW (Rogier et al., 2019; Velotti et al., 2020). Notably, although not examined in our research, compared with the cisgender MSM population, transgender MSM report low levels of identity concealment, but are more likely to engage in depression and HIV-related sexual behaviors, which play a considerable role in new HIV infections in China (Storm et al., 2021). Providing targeted psychosocial interventions and open, tolerant social environment for different gender minorities within the MSM population is equally crucial (Duan et al., 2021).

Several limitations were noted in this study. First, the concealment of the MSM population precludes traditional randomized sampling methods, although MSM with different types of characteristics were recruited based on the pre-survey results, attentions should be paid to the possible selections bias when generalizing our findings to the target MSM population. Second, anxiety, depression, and other variables were measured by self-reported questionnaire, the results for some sensitive issues may therefore be biased. Third, age was a significantly different variable in depression and the stratified analysis of age groups could be conducted in subsequent studies. Lastly, limited by the overall project, only HIV-negative MSM were recruited in the current study, follow-up studies may consider expanding the sample population to people living with HIV (PLWH).

## Conclusion

Our study on anxiety and depression in MSM showed that the development trajectories of anxiety and depression had heterogeneity. The prevalence of anxiety and depression was high. Demographic characteristics, psychosocial characteristics, HIV-related knowledge, attitude, and behavior all affected the development of anxiety and depression. In terms of key populations, it is necessary to prevent and remedy mental health issues among MSM with low literacy, history of STDs and bisexuality, and provide timely and effective psychological counseling; in terms of interventions, it is necessary to reduce social discrimination by increasing social support for the MSM, including the establishment of a MSM community and strengthening the professionalism of healthcare personnel, while at the same time providing appropriate behavioral interventions for the MSM, popularizing HIV health education, and promoting safe sex to avoid the development of anxiety and depression in the MSM.

## Data availability statement

The original contributions presented in this study are included in the article/supplementary material, further inquiries can be directed to the corresponding author.

## Ethics statement

The studies involving human participants were reviewed and approved by the Ethics Committee of Chongqing Medical University. The patients/participants provided their written informed consent to participate in this study.

## Author contributions

AH designed the cohort study and guided research implementation. XZ, JD, and HL administered the project. HP, YG, BL, and XT performed the experiments, supervised the execution of the study, and checked the quality of data. DW and RD wrote the manuscript. XZ, DW, and XT revised the manuscript. All authors contributed to the article and approved the submitted version.
